# Pimobendan prevents cardiac dysfunction, mitigates cardiac mitochondrial dysfunction, and preserves myocyte ultrastructure in a rat model of mitral regurgitation

**DOI:** 10.1186/s12917-023-03693-2

**Published:** 2023-08-23

**Authors:** Pakit Boonpala, Nakkawee Saengklub, Sirinapa Srikam, Wilawan Ji-au, Yaowalak Panyasing, Sarawut Kumphune, Anusak Kijtawornrat

**Affiliations:** 1 Department of Physiology, Faculty of Veterinary Science, Chulalongkorn University, Bangkok, Thailand; 2 Chulalongkorn University Laboratory Animal Center, Chulalongkorn University, Bangkok, Thailand; 3 Department of Physiology, Faculty of Pharmacy, Mahidol University, Bangkok, Thailand; 4 Department of Pathology, Faculty of Medicine, Chulalongkorn University, Bangkok, Thailand; 5 Department of Pathology, King Chulalongkorn Memorial Hospital, Bangkok, Thailand; 6 Department of Pathology, Faculty of Veterinary Science, Chulalongkorn University, Bangkok, Thailand; 7 Biomedical Engineering Institute (BMEI), Chiang Mai University, Chiang Mai, Thailand; 8 Biomedical Engineering and Innovation Research Center, Chiang Mai University, Chiang Mai, Thailand

**Keywords:** Mitochondrial quality, Mitral regurgitation, Pimobendan, Rat, Reactive oxygen species

## Abstract

**Background:**

Pimobendan has been proven to delay the onset of congestive heart failure (CHF) in dogs with mitral regurgitation (MR); however, molecular underlying mechanisms have not been fully elucidated. This study aimed to investigate (1) the effects of pimobendan on cardiac function, cardiac mitochondrial quality and morphology, and cardiac ultrastructure in a rat model of chronic MR and (2) the direct effect of pimobendan on intracellular reactive oxygen species (ROS) production in cardiac cells. MR was surgically induced in 20 Sprague-Dawley rats, and sham procedures were performed on 10 rats. Eight weeks post-surgery, the MR rats were randomly divided into two groups: the MR group and the MR + pimobendan group. Pimobendan (0.15 mg/kg) was administered twice a day via oral gavage for 4 weeks, whereas the sham and MR groups received equivalent volumes of drinking water. Echocardiography was performed at baseline (8 weeks post-surgery) and at the end of the study (4 weeks after treatment). At the end of the study protocol, all rats were euthanized, and their hearts were immediately collected, weighed, and used for transmission electron microscopy and mitochondrial quality assessments. To evaluate the role of pimobendan on intracellular ROS production, preventive or scavenging properties were tested with H_2_O_2_-induced ROS generation in rat cardiac myoblasts (H9c2).

**Results:**

Pimobendan preserved cardiac functions and structure in MR rats. In addition, pimobendan significantly improved mitochondrial quality by attenuating ROS production and depolarization (*P* < 0.05). The cardiac ultrastructure and mitochondrial morphology were significantly preserved in the MR + pimobendan group. In addition, pimobendan appeared to play as a ROS scavenger, but not as a ROS preventer, in H_2_O_2_-induced ROS production in H9c2 cells.

**Conclusions:**

Pimobendan demonstrated cardioprotective effects on cardiac function and ultrastructure by preserving mitochondrial quality and acted as an ROS scavenger in a rat model of MR.

**Supplementary Information:**

The online version contains supplementary material available at 10.1186/s12917-023-03693-2.

## Background

Degenerative mitral valve disease (DMVD) is the most common heart disease in dogs responsible for approximately 75% of heart disease cases visiting veterinary practitioners worldwide, especially in North America [1]. In DMVD, the mitral valve does not close properly, causing blood to flow backward into the left atrium from the left ventricle as it contracts. This disease initiates volume overload, leading to several subsequent compensatory mechanisms [2]. Ultimately, these compensatory adjustments weaken the heart and lead to decompensated mitral regurgitation (MR) (i.e., heart failure). In recent prospective studies from randomized clinical trials in the field of veterinary cardiology, pimobendan administration delays the onset of congestive heart failure (CHF) by a median of 15 months in dogs with DMVD stage B2 [3]. Moreover, adding pimobendan to heart failure therapy in dogs with furosemide and angiotensin-converting enzyme inhibitors delayed the onset of refractory signs of heart failure, improved clinical conditions, and increased survival time [4–6]. Although pimobendan inhibits phosphodiesterase-III (PDE-III) causing an increase in cyclic adenosine monophosphate (cAMP) and sensitizes the cardiac contractile apparatus (i.e., troponin C) to intracellular calcium, the definite mechanism of pimobendan underlying the delay in the onset of CHF or refractory signs of CHF is still unclear.

Cardiac mitochondrial dysfunction has been demonstrated to play a crucial role in HF and is believed to contribute to the progression of HF through both diminished high-energy phosphate production and amplified production of reactive oxygen species (ROS), resulting in high levels of oxidative stress [7, 8]. The intracellular ROS generated by damaged or malfunctional mitochondria also causes mitochondrial dysfunction, which initiates cardiac remodeling and damages sarcomeric proteins [9]. Furthermore, stretched myocytes produce ROS, leading to cardiomyocyte degeneration, and are associated with contractile dysfunctions in the pathophysiology of HF [10–12]. Moreover, ROS have been found to trigger cardiac mitochondrial membrane potential changes [13]. Although several PDE-III inhibitors mitigate ROS-induced mitochondrial dysfunction [14], the role of pimobendan in cardiac mitochondrial function has never been investigated.

In this study, MR was surgically induced in rats to test the hypothesis that pimobendan (PIMO) can prevent cardiac dysfunction and mitigate MR-induced cardiac mitochondrial dysfunction by reducing ROS production, preventing mitochondrial depolarization, and preserving myocyte ultrastructure. The direct effect of pimobendan on H_2_O_2_-induced oxidative stress generation in H9c2 cells was also investigated.

## Results

### Pimobendan prevents cardiac dysfunction

**Fig. 1:**
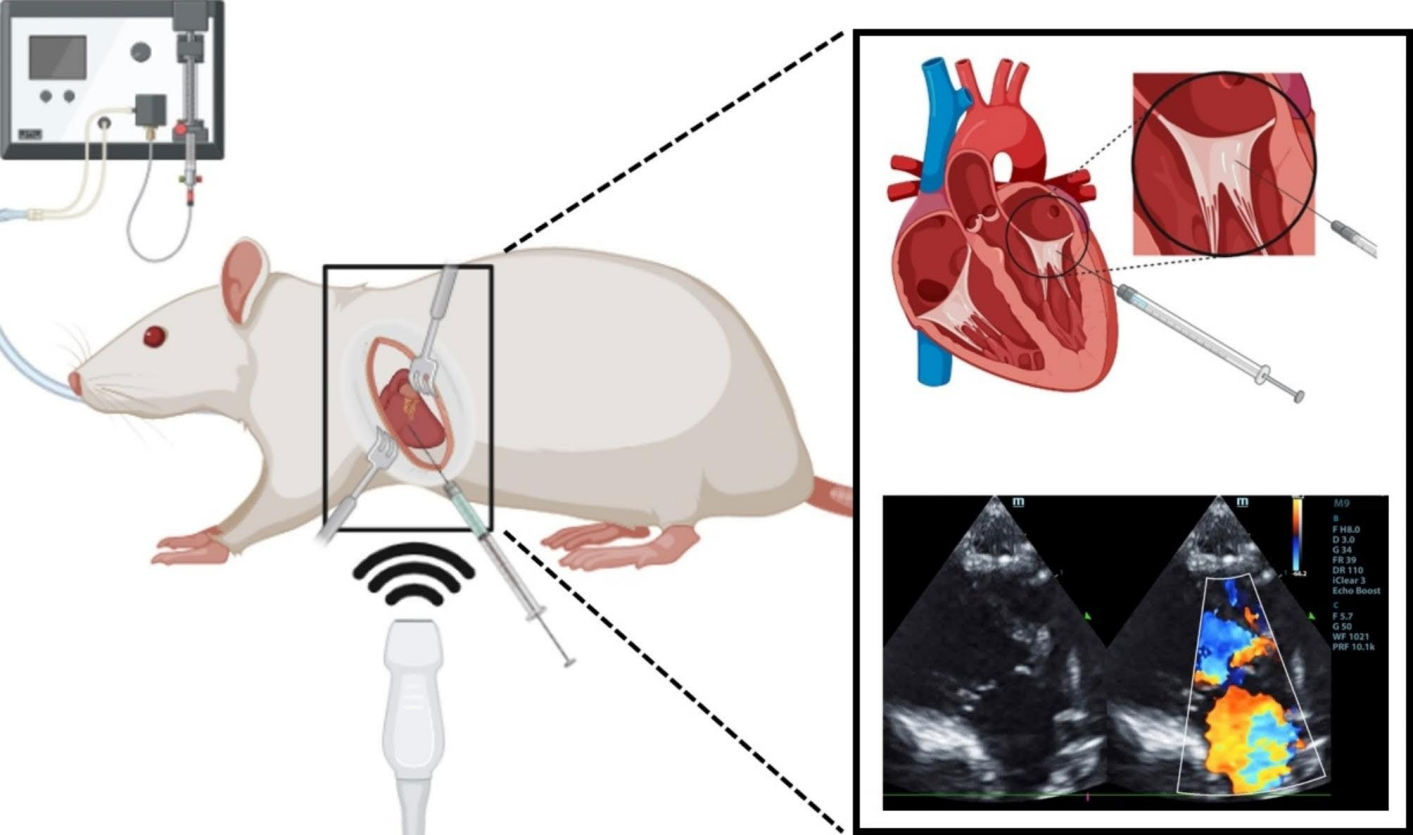
Establishment of mitral regurgitation (MR) in rats. (Left and upper right) MR creation using a needle puncture through the left ventricular free wall, and MR was confirmed by echocardiography. (Lower right) Right parasternal long-axis color Doppler echocardiography view demonstrating normal blood flow across the mitral valve and backward mitral regurgitant jet into the left atrium immediately after the puncture (mosaic pattern) (Figure created with BioRender.com).

The effect of pimobendan on cardiac geometry and left ventricular **(**LV**)** function in MR-induced left atrial **(**LA**)** and LV dilatation and LV dysfunction was investigated by echocardiography. After the puncture through the mitral valve leaflets, the regurgitant jet was documented by Doppler echocardiography as a mosaic pattern of jet flow backward into the left atrium **(**Fig. 1**).** Similar MR jet areas in MR and MR + PIMO rats were found at baseline and after 4 weeks of oral administration of placebo and pimobendan, respectively. Eight weeks after surgery, the LV **(**i.e., left ventricular internal diameter at end-diastole, LVIDd; left ventricular internal diameter at end-systole, LVIDs**)** in MR and MR + PIMO rats appeared to be increased but did not reach statistical significance when compared with sham rats (Supplement Tables 1 and 2). At 12 weeks **(**i.e., 4 weeks post-treatment**)**, both LVIDd and LVIDs of the MR rats continued to increase and became significantly enlarged when compared with the sham rats (LVIDd MR:0.99 ± 0.04 cm vs. sham: 0.80 ± 0.01 cm, *P* < 0.001; LVIDs MR: 0.66 ± 0.04 cm vs. sham: 0.49 ± 0.03 cm, *P* = 0.003**)**. However, pimobendan prevented the increase in LVIDd and LVIDs diameters (LVIDd MR + PIMO: 0.81 ± 0.04 cm, *P* < 0.001; LVIDs MR + PIMO: 0.43 ± 0.03 cm, *P* < 0.001) so that they significantly decreased when compared with the MR rats but did not differ from the sham rats. Eight weeks after surgery, the ejection fraction **(**EF**)** did not differ among groups (sham: 70.86 ± 2.76**%**, MR: 76.49 ± 2.39**%**, MR + PIMO: 76.29 ± 1.46**%**; P = 0.158); however, 4 weeks after treatment, the EF of MR + PIMO was significantly higher when compared with the MR group at the same timepoint **(**MR + PIMO: 82.71 ± 1.85**%** vs. MR:66.79 ± 2.78**%**; *P* = 0.001). At the end of the study, the end-diastolic volume **(**EDV**)** and end-systolic volume **(**ESV**)** of the MR rats were significantly higher than those of the sham rats **(**EDV MR: 2.07 ± 0.20 mL vs. sham: 1.12 ± 0.05 mL, *P* < 0.001; ESV MR: 0.70 ± 0.11 mL vs. sham: 0.31 ± 0.05 mL, *P* = 0.003**)** while the EDV and ESV of the MR + PIMO groups **(**EDV 1.20 ± 0.13 mL, ESV 0.22 ± 0.04 mL, *P* < 0.001 and *P* < 0.001, respectively**)** were significantly lower than those of the MR rats, suggesting that the LV dilates in MR groups and pimobendan prevents it. While no significant change in heart rate was found among the groups both at baseline **(**sham: 329.0 ± 14.9 bpm; MR: 359.2 ± 6.2 bpm; MR + PIMO: 345.7 ± 12.9 bpm; *P* = 0.219**)** and end of the study **(**sham: 344.9 ± 12.2 bpm; MR: 347.5 ± 10.5 bpm; MR + PIMO: 366.7 ± 12.6 bpm; *P* = 0.374**)**, the cardiac output **(**CO**)** increased significantly in MR rats when compared with sham rats **(**Baseline MR: 0.42 ± 0.04 L/min vs. sham: 0.24 ± 0.02 L/min; P < 0.001; End of the study MR: 0.48 ± 0.04 L/min vs. sham: 0.29 ± 0.02 L/min; *P* = 0.001**).** Since HR did not change, this increase in CO is also directly attributable to the LVIDd and LVIDs changes, suggesting adaptive eccentric hypertrophy due to volume overload.

**Fig. 2:**
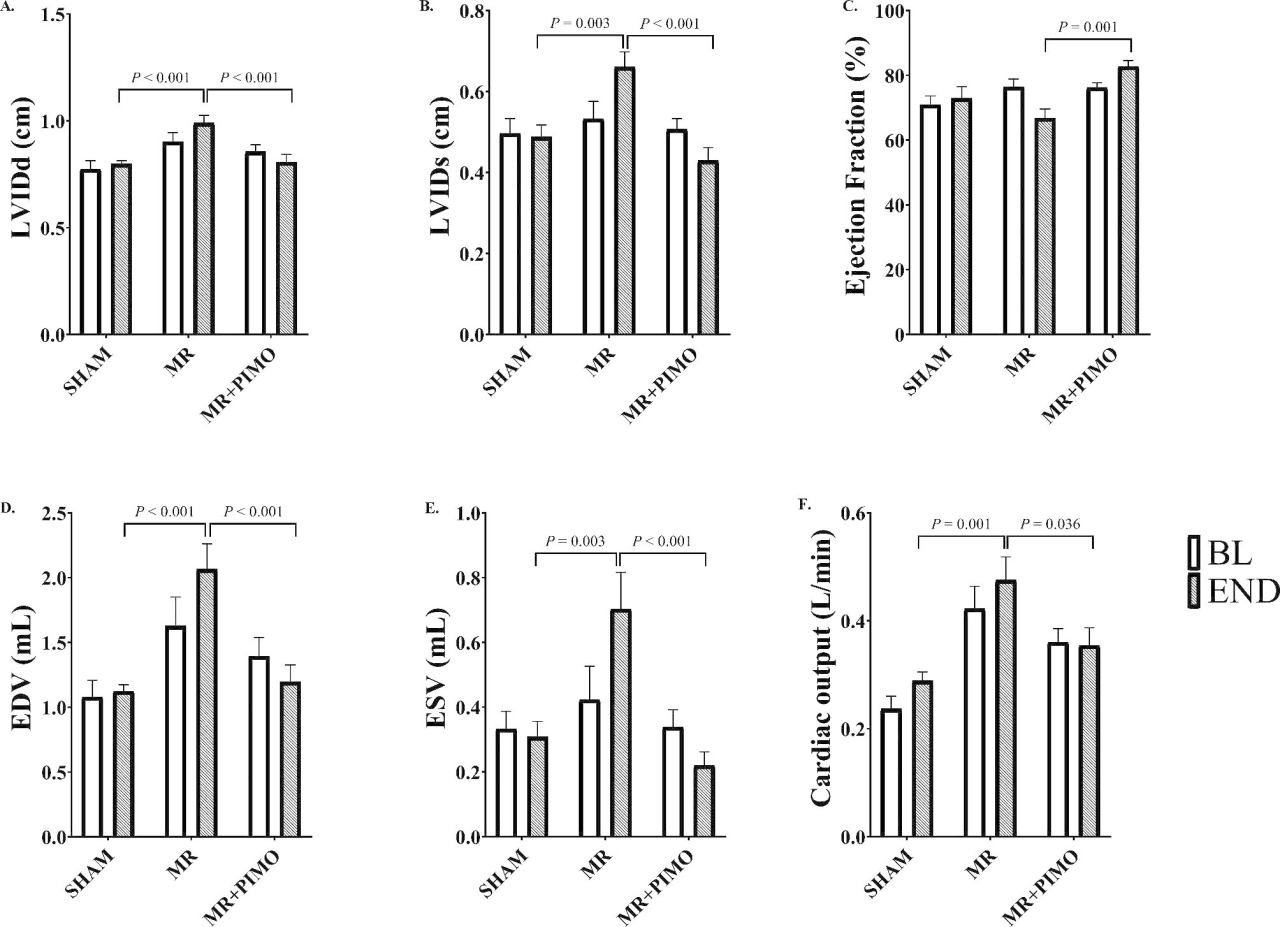
Pimobendan **(**PIMO**)** prevents mitral regurgitation **(**MR**)**-induced myocardial dysfunction assessed by echocardiography. **(**A**)** Left ventricular internal diameter at end-diastole **(**LVIDd**)**, **(**B**)** left ventricular internal diameter at end-systole **(**LVIDs**)**, **(**C**)** ejection fraction **(**EF**)**, **(**D**)**, end-diastolic volume, **(**E**)** end-systolic volume, and **(**F**)** cardiac output **(**CO**)** are shown for each group. Data are presented as mean ± standard error of mean **(**SEM**)**. Two-way ANOVA with Tukey’s post hoc test for the analysis of group differences. *P < 0.05 for differences among groups and between timepoints. BL = At the baseline; End = At the end of the experiment

### Pimobendan stabilizes cardiac mitochondrial quality

**Fig. 3:**
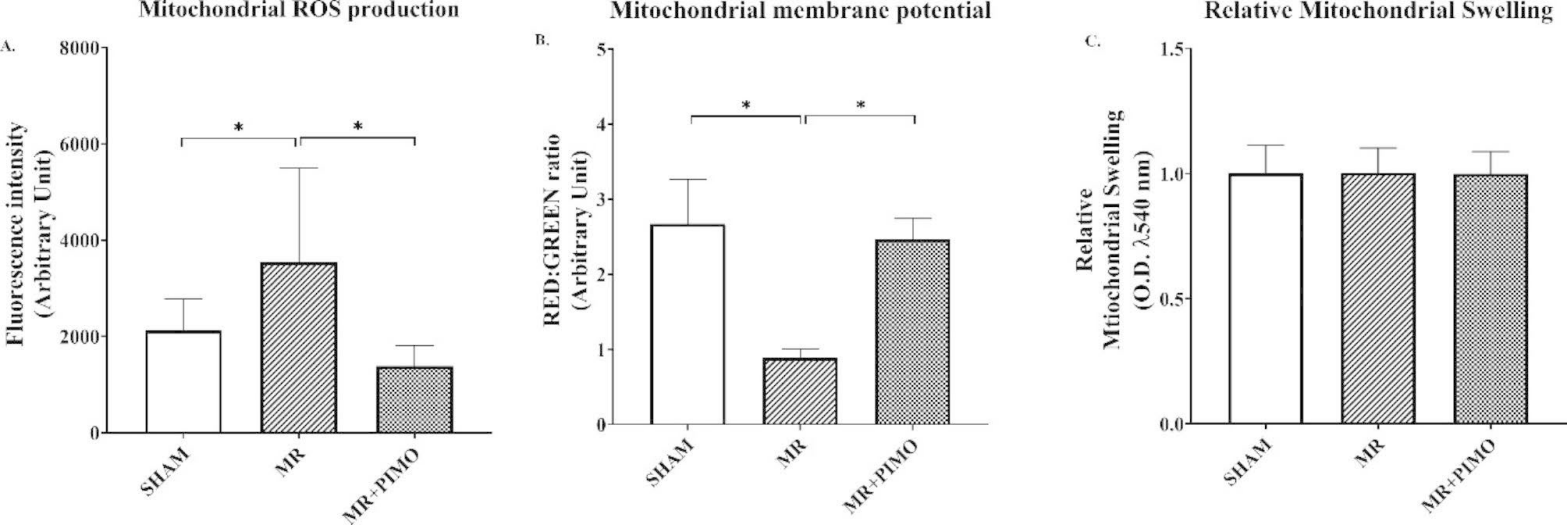
Effects of pimobendan on mitochondrial quality. **(**A**)** Fluorescent intensity of reactive oxygen species **(**ROS**)** production in LV mitochondrial tissue. **(**B**)** Assessment of mitochondrial membrane potential change by fluorescence intensity of the red:green ratio. **(**C**)** Cardiac mitochondrial swelling. Variables are presented as mean ± standard error of mean **(**SEM**)**. Differences among groups **(**sham, *n* = 10; MR, *n* = 10; pimobendan, *n* = 10**)** were compared using a one-way analysis of variance followed by Tukey’s post hoc test for multiple comparisons. *Significant difference **(***P* < 0.05**)** among the groups

This study demonstrated that MR-producing volume overload resulted in cardiac mitochondrial dysfunction suspected to be attributable to excessive mitochondrial ROS production and mitochondrial depolarization **(**i.e., reduced red/green fluorescence intensity**)** when compared with those in sham rats **(***P* < 0.05, Fig. 3A and B**).** The results showed no change in the absorbance of mitochondria, which indicated no alteration in mitochondrial swelling among the groups **(**Fig. 3C). Pimobendan significantly reduced mitochondrial ROS production and increased red:green fluorescence intensity **(***P* < 0.05**)**.

### Pimobendan preserves the myocyte ultrastructure

**Fig. 4:**
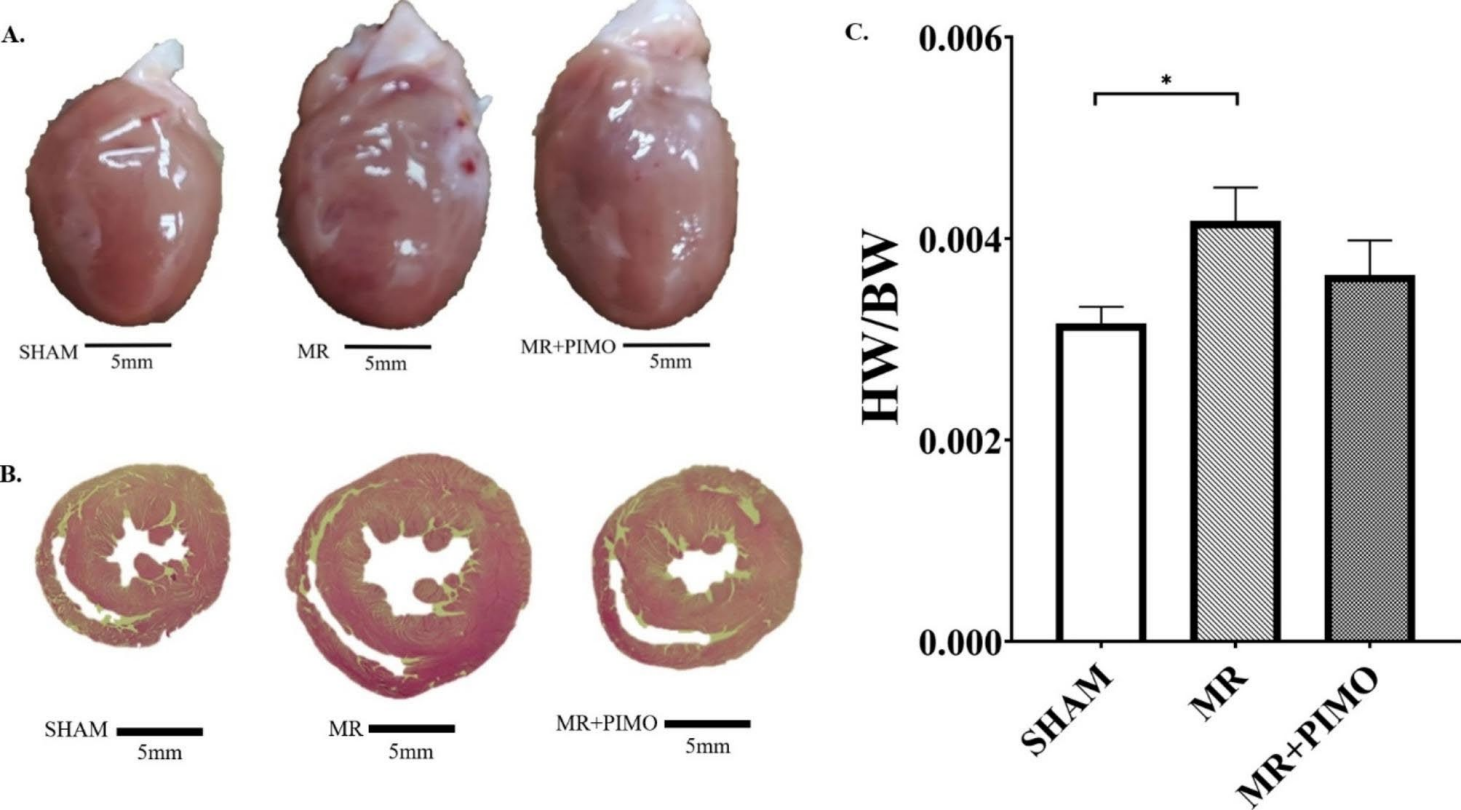
Representative images of the whole heart (A) and mid-cardiac cross-sectional morphology from hematoxylin and eosin **(**H&E**)** staining (B) in the sham (left), mitral regurgitation (MR; middle), and mitral regurgitation + pimobendan (MR + PIMO; right) rats. Notice the left ventricular chamber enlargement in the MR. Heart-to-bodyweight ratio analysis (C). Data are presented as mean ± standard error of the mean **(**SEM**)**. Differences between the groups (sham, *n* = 10; MR, *n* = 10; pimobendan, *n* = 10) were compared using one-way analysis of variance followed by Tukey’s post hoc test for multiple comparisons. * Significant difference (*P* < 0.05) among the groups

**Fig. 5:**
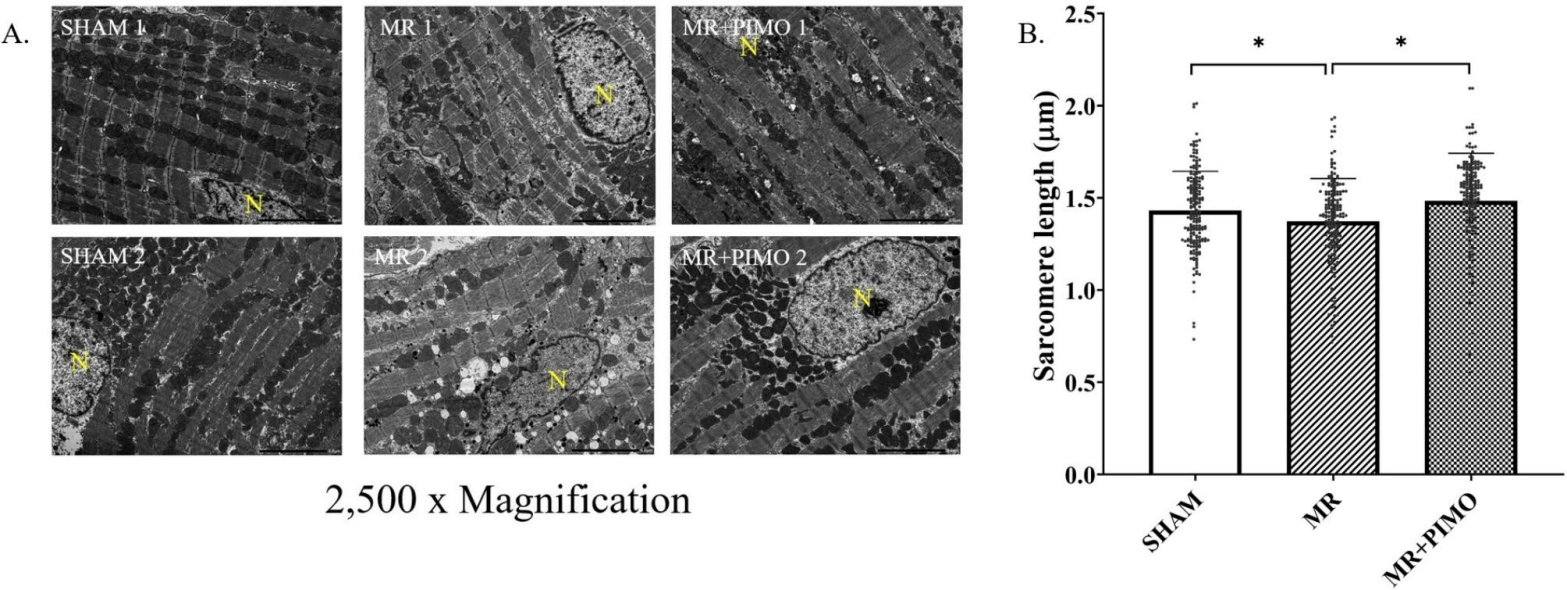
Transmission electron microscopy (TEM) images detailing the sarcomere structure, intracellular organelles, and their spatial organization. (A) Sarcomere structure from two different rats per group, depicting an organized sarcomeric network, with clearly visible and condensed Z-line, I-band, and A-band. The nucleus (N) is labeled in yellow in each image. A linear registry of mitochondria in sham rats is interspersed parallel to the myofibrils and adjacent to the sarcomeres (left column). After 12 weeks of mitral regurgitation (MR), ultrastructural changes are evident. The I-band was not apparent, and the density of the Z-band was reduced. The mitochondria interspersed between the sarcomeres were fragmented and disorganized. Vacuoles were overrepresented in one of the MR rats **(**MR 2**).** In MR treated with pimobendan for 4 weeks, damage to the sarcomeres was less evident compared with MR rats treated with placebo. (B) Sarcomere length measured from TEM images of the sham, MR, and MR + pimobendan (MR + PIMO**)** rats. Data are presented as mean ± standard error of the mean (SEM). The differences between groups (sham, *n* = 10; MR, *n* = 10; pimobendan, *n* = 10) were compared using a one-way analysis of variance, followed by Tukey’s post hoc test for multiple comparisons. * Significant difference (*P* < 0.05) among the groups

**Fig. 6:**
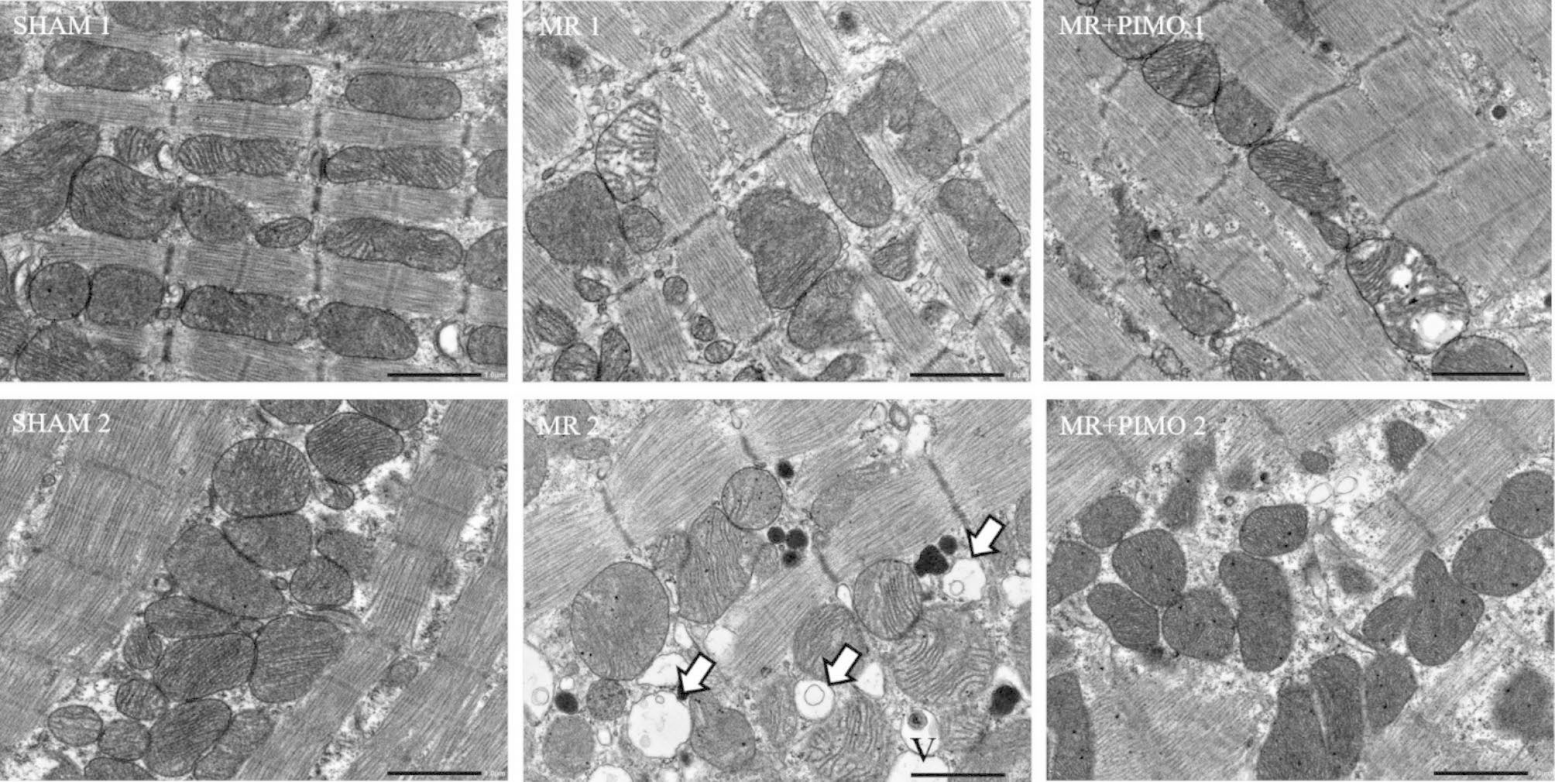
Transmission electron microscopy (TEM) images (10,000 magnification) detailing the mitochondrial organization and presence of vacuoles in the left ventricular trabeculae muscle from the sham, MR, and MR + PIMO groups. Representative images from two individual samples from different rats are shown in the sham, MR, and MR + PIMO groups. The vacuoles are depicted by white arrows in the images. Sham and MR + PIMO rats had fewer vacuoles present within the myocardium. A higher presence of vacuoles was noted in MR rat no. 2

The gross morphology of the heart together with the LV cross-section was compared among the three groups (Fig. 4A and B). In addition, the heart weight per bodyweight ratio was presented (Fig. 4C). When compared among the groups, the hearts with MR appeared to be larger than that of sham and MR + PIMO rats which supported the echocardiographic findings. The LV chamber was also dilated in MR rats when compared with sham rats. The heart weight to body weight ratio was significantly increased in MR rats (MR:0.0042 ± 0.0003 vs. sham: 0.0031 ± 0.0002; P = 0.038), and pimobendan tended to reduce that ratio (MR + PIMO: 0.0036 ± 0.0003 *P* = 0.391). High-resolution transmission electron microscopy **(**TEM**)** of the myocyte sarcomeric structure is shown in Fig. 5A. In sham rats, the sarcomeric structure was normal and composed of the A-band, I-band, M-line, and Z-line. Substantial loss of the sarcomeric structure in MR and MR + PIMO was found, but more in MR rats. Myocytes from MR rats demonstrated a higher density of mitochondria surrounding and interspersing the sarcomeres and substantially more clustering around the nucleus than MR + PIMO rats. The sarcomeric length was significantly lower in MR rats than in sham rats; however, pimobendan preserved the sarcomeric length (Fig. 5B). The mitochondrial organization within the myocytes is shown in Fig. 6. In sham rats, the mitochondria were aligned as a linear registry along the sarcomeres, with some clustering around myofibrils. In myocytes from MR rats, the mitochondria were enlarged with irregular and disorganized cristae, and large vacuoles were observed. The increase in the matrix space was also present in MR rats more than in MR rats treated with pimobendan. Focal areas of myocytolysis and fragmentation of myofibrils were observed more in MR rats than in MR + PIMO rats. In MR rats receiving pimobendan, the amount of mitochondria clustering and interspersing the sarcomeres are less than that in MR rats. The number and size of vacuoles were also smaller than those of MR rats.

### Effect of pimobendan on H_2_O_2_-induced severe oxidative stress in H9c2 cells

**Fig. 7:**
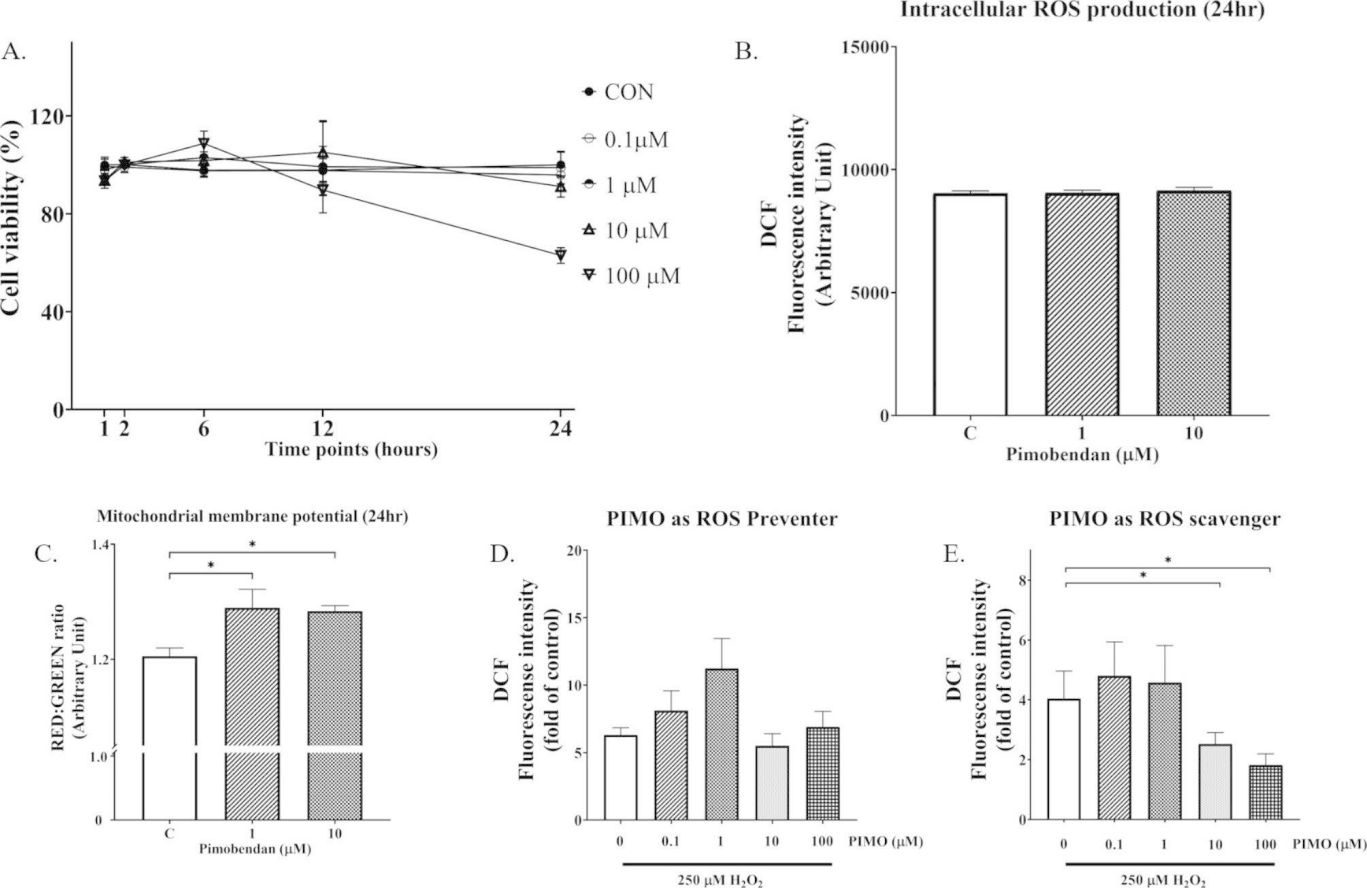
Effect of pimobendan **(**PIMO**)** on the viability of H9c2 cells and effects of pimobendan on H_2_O_2_-induced reactive oxygen species **(**ROS**)** in H9c2 cells. **(**A**)** Pimobendan decreased the viability of H9c2 cells in a concentration- and time-dependent manner. Effects of pimobendan on cellular ROS production **(**B**)** and mitochondrial membrane potential change **(**C**).** Fluorescent intensity of intracellular ROS analysis by dichloro-dihydro-fluorescein diacetate (DCFDA) in H9c2 cells exposed to 250 µM H_2_O_2_ when pimobendan was used as an ROS preventer (D) and as an ROS scavenger (E). Values (mean ± SEM) are presented as the fold changes in fluorescence relative to untreated cells. Each assay was completed at least six times, with two biological replicates each. Statistical analysis was performed by one-way analysis of variance, followed by Tukey’s multiple comparison test. * *P* < 0.05

Treatment with several concentrations of pimobendan **(**0, 0.1, 1, and 10 µM**)** did not result in significant changes in cellular viability at any of the incubation times **(**1, 2, 6, 12, and 24 h**)** (Supplement Table 3). However, treatment of 100 µM pimobendan for 12 and 24 h could reduce cell viability lower than 90**% (**LD10**) (**89.68 ± 1.34**%** and 62.92 ± 1.32**%**, respectively**) (**Fig. 7A). Therefore, the optimum condition for treatment in this experiment was 10 µM pimobendan for 24 h, which was used for the subsequent protocol in this study.

To investigate the effect of pimobendan on intracellular ROS production and mitochondrial membrane potential change in cultured H9c2 cells, pimobendan at 1 and 10 µM was tested for its effects, and the results are shown in Fig. 7B C. Both concentrations of pimobendan did not affect intracellular ROS production. However, pimobendan at 1 and 10 µM could reduce mitochondrial depolarization.

To determine the ability of pimobendan to prevent intracellular ROS production in the H_2_O_2_-induced oxidative stress model on H9c2 cells, several concentrations of pimobendan were evaluated. As shown in Fig. 7D, all concentrations of pimobendan did not affect the ROS production.

In addition, the effect of pimobendan as a ROS scavenger in which the H_2_0_2_ was mixed with pimobendan and incubated for 30 min before adding to H9c2 cells was evaluated, and then ROS production was measured. The results showed that the level of ROS production was lower in cells exposed to a mixture of 10 or 100 µM pimobendan plus H_2_O_2_ than in the control group without pimobendan. Therefore, pimobendan could reduce the ability of H_2_O_2_ to induce intracellular ROS production in H9c2, suggesting a scavenging effect of pimobendan (Fig. 7E).

## Discussion

To investigate the potential cardioprotective effect of pimobendan on MR-induced LV dysfunction and mitochondrial dysfunction, we surgically induced MR by puncturing the mitral valve with a 20-G needle through the LV free wall as shown in Fig. 1. Marked cardioprotective effects of pimobendan were found on the rodent model of MR-induced HF associated with cardiac mitochondrial quality preservation and ROS scavenging. The main results are as follows: (1) MR-induced LV dysfunction and cardiac remodeling and subsequently HF; (2) MR induced mitochondrial dysfunction by increasing ROS production and mitochondrial depolarization; and (3) pimobendan attenuated LV dysfunction and remodeling, preserved cardiac mitochondrial quality, and acted as an ROS scavenger. To our knowledge, this is the first study proposing the underlying mechanism of pimobendan to delay the onset of MR-induced HF. In addition, we proposed a potential mechanism to explain the effect of pimobendan in preventing cardiac dysfunction that may be due to (1) a direct ROS scavenger effect inferred from the effect of pimobendan on H_2_O_2_-induced ROS production and (2) pimobendan attenuated mitochondrial ROS (mtROS) production, which in turn reduces the mitochondrial membrane potential change thereby reducing cellular injury.

Our results support the hypothesis that pimobendan not only increased systolic function but also potentially improved diastolic function. This is supported by our data that shows significant attenuation of mitochondrial dysfunction (Figs. 3, 5 and 6). Cardiac contraction and relaxation require a large amount of adenosine triphosphate (ATP) supplied by healthy mitochondria. In HF, the mitochondria are dysfunctional resulting in impaired myocardial energetics (e.g., decreased ATP content) subsequently less ATP available for muscular relaxation [15]. Several studies in dogs [16–18] have shown improvement of diastolic function by pimobendan which could be partly explained by our findings.

MR remains a crucial problem in mature, small–medium canines worldwide. Immediately after mitral valve leakage, compensatory mechanisms (e.g., renin angiotensin aldosterone system and sympathetic nervous system) are activated to compensate for the reduced volume of blood ejected into the aorta. These compensatory mechanisms produced by the Frank–Starling mechanism are good for the heart to generate adequate perfusion pressure; however, this maladaptation leads to cardiac remodeling and later progresses to a compensatory phase in which CHF develops. In veterinary medicine, pimobendan is the only drug that can delay CHF onset when used in the appropriate stage of DMVD (i.e., DMVD stage B2) [1]. However, the underlying molecular mechanism has not been fully elucidated. This may be because of the difficulties in obtaining cardiac samples from client-owned canine patients; thus, small animal models of HF are needed. In the present study, we surgically induced MR to mimic the pathophysiology of MR in dogs and obtained cardiac muscles for molecular investigation. Previous reports [19–21] show that MR rats possess similar responses to MR dogs in which volume overload induced hypercontractility (i.e., 8 weeks after operation) as shown by increased EF and LV remodeling inferred from increased left ventricular internal diameter. In the later phase (12 weeks from surgery), impaired LV contractility develops similar to that observed in MR dogs; thus, the MR rat provides a model to investigate the molecular mechanisms and response to interventions. However, the echocardiographic parameters at 8 weeks post-surgery of our MR rats did not show statistically significant differences when compared with the sham rats.

Pimobendan, a benzimidazole-pyridazinone derivative, so-called inodilator, acts by inhibiting PDE-III leading to increased concentrations of cAMP and sensitizing the cardiac contractile apparatus to intracellular calcium, producing vasodilation and positive inotropic effects that have been demonstrated in several species [18, 22–25]. This study showed that pimobendan maintained cardiac contractility through the end of the experiment. In addition to the positive inotropic effect, our results revealed a reverse remodeling effect of pimobendan inferred from a reduction in both LVIDd and LVIDs. These effects were also demonstrated in clinical trials [3]. In addition, the finding of pimobendan on reverse remodeling may support the pharmacologic annuloplasty hypothesis in which pimobendan decreases severity of MR due to less LV remodeling and subsequently less mitral annular dilatation. It has been postulated that progressive LV enlargement alters ventricular geometry and annular dilatation causing increased severity of MR [26]. Several medicines (i.e., sacubitril/valsartan, carvedilol, losartan) have been reported to possess reverse remodeling effects [27], with long-standing treatment, resulting in less LV remodeling and subsequently less mitral annular dilatation. Pimobendan has been shown to reduce severity of MR for both short-term and long-term treatment [3, 28]. In short-term treatment with pimobendan, it has been demonstrated recently in dogs with DMVD stage B1 and B2 that the regurgitant fraction was reduced after receiving pimobendan for 7–10 days [28].

Previous studies have shown that stretching resulting from chronic volume overload leads to increased ROS production by the mitochondria causing cytoskeletal disruption preceding left ventricular systolic failure [29–31]. ROS, small molecules, are produced as a result of the normal aerobic metabolism in mitochondria [32]; ROS in low concentrations act as secondary messengers, whereas high concentrations cause oxidative stress and damage [33] to cells and mitochondria, which is responsible for several pathological diseases [34, 35]. This study demonstrated that mtROS production and mitochondrial depolarization were increased in MR rats and that treatment with pimobendan protects against these changes. The possible mechanism responsible for this finding is the cardioprotective properties of PDE-3i of pimobendan, and this is supported by a previous study reporting that PDE-III inhibitors (i.e., cilostazol) attenuated oxidative stress-induced mitochondrial dysfunction in the isolated cardiac mitochondria from rat hearts [14, 36]. Another possibility is that PDE-III inhibitors can activate cAMP-dependent protein kinase A and potentiate the opening of mitochondrial calcium-activated potassium channels, which had been demonstrated previously in a rabbit model of myocardial infarction by Fukasawa et al. [37]. Furthermore, our study showed that pimobendan can act as an ROS scavenger by reducing ROS production in H9c2 cells induced by H_2_O_2_.

In this study, we also explored ultrastructural changes and measured sarcomeric length in the myocytes of a rodent model of MR. In MR rats, a reduction in sarcomeric length was observed in this study similar to previous reports [38, 39], which could be due to the breakdown of structural organization, especially the thin filament [38]. In addition, MR causes a loss of the linearity of the mitochondria along the sarcomeres, clustering around the nucleus and sarcomeres, and abnormalities in the cristae. Studies have reported that these structural changes are associated with mitochondrial dysfunction [31] and may contribute to the development of oxidative stress [38, 40, 41], which supports our findings of mitochondrial quality degradation in MR. Furthermore, in the current manuscript, we demonstrated the presence of vacuoles in the heart of MR rats more than in the heart of treatment groups or sham rats. Mitochondrial vacuolization is regarded as a qualitative indicator of mitochondrial injury. In this study, the presence of mitochondrial vacuoles confirms the occurrence of mitochondrial injury in the MR model, in addition to mitochondrial ROS production and alterations in mitochondrial membrane potential. Further study to quantify this change in mitochondrial vacuoles is warranted.

This study had several limitations. Pimobendan possesses both calcium sensitizing and PDE-III inhibiting effects, which are involved in many signaling pathways that can enhance cardiac and vascular functions in heart failure. This study investigated only the role of pimobendan in cardiac mitochondrial quality and used the mitochondrial morphology to support the findings of mitochondrial quality. Moreover, many mechanisms of regulated cell death (e.g., apoptosis, necroptosis, pyroptosis, and ferroptosis) contribute to HF. We did not investigate the role of pimobendan in these mechanisms. Further studies are needed to determine the effect of pimobendan on these signaling pathways and programmed cell death.

**Fig. 8:**
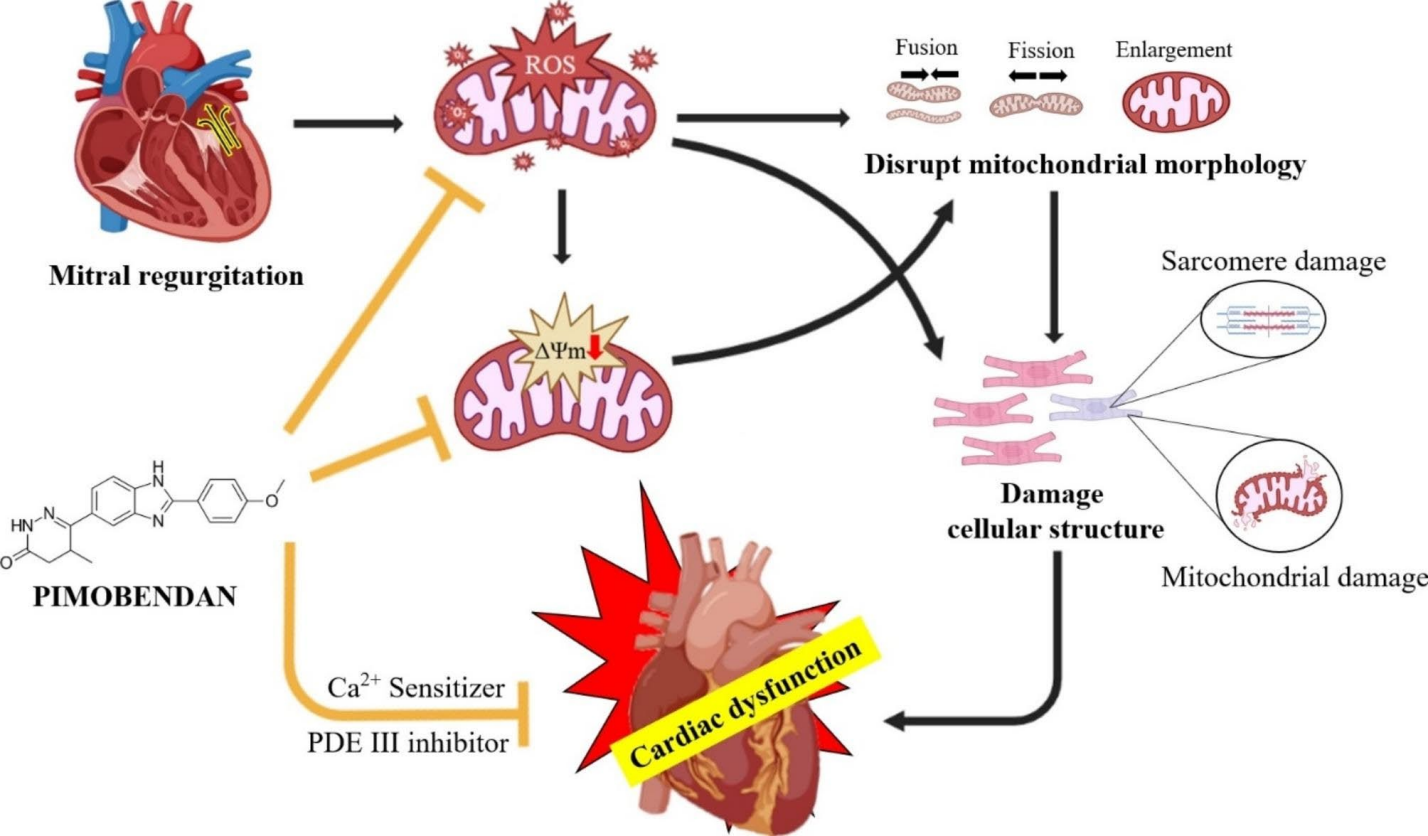
Pimobendan prevents left ventricular dysfunction by attenuating mitochondrial ROS production and mitochondrial depolarization as evidenced by the maintenance of cellular ultrastructure and mitochondria morphology

In conclusion, pimobendan slowed cardiac remodeling as shown by echocardiography and histopathological findings as well as helped to preserve function as shown by EF (Figs. 2 and 4). Pimobendan preserved cardiac mitochondrial quality, inferred from a reduction in mtROS production and mitochondrial depolarization (Fig. 8). In addition, pimobendan has a direct effect at the cellular level as an ROS scavenger. This study also demonstrated evidence of the cardioprotective effect of pimobendan by the preservation of the myocyte ultrastructure and mitochondrial morphology observed under TEM. These findings may help explain its effect on delaying heart failure observed in MMVD dogs.

## Methods

### Animals

This study was approved by the Institutional Animal Care and Use Committee of Chulalongkorn University Laboratory Animal Center (Animal Use Protocol No. 1873022). Thirty male Sprague-Dawley rats, weighing 300–350 g, were obtained from Nomura Siam International Co., Ltd. All rats were housed in an individual ventilated cage at the Chulalongkorn University Laboratory Animal Center. The rats were allowed to access autoclavable commercial feed and water ad libitum. The light/dark cycle was set at 12:12 h, and the temperature and humidity were kept constant at 22 °C ± 1 °C and 50% ± 10%, respectively. All experimental procedures in animals were performed in accordance with the Guide for the Care and Use of Laboratory Animals [42] and Animal Welfare Act 2015. All reported methods are in accordance with the ARRIVE 2.0 guidelines.

### Induction of mitral regurgitation

In an anesthetic chamber, all rats were anesthetized with isoflurane, followed by orotracheal intubation. Rats were ventilated using rodent ventilation with 2.5 mL of tidal volume, and the ventilatory rate was set at 80 breaths per min (Harvard Apparatus, MA, USA). The isoflurane was maintained between 2.0% and 2.3% with 100% oxygen. After hair clipping on the left thorax, the area was prepared aseptically. Perioperative analgesia and antibiotics included tramadol (12.5 mg/kg, intraperitoneal route [IP]) and diluted enrofloxacin (10 mg/kg, subcutaneous route [SC]), which were given to alleviate surgical pain and reduce the risk of bacterial infection, respectively. The process of surgery-induced MR was described previously [20]. Briefly, the incision was made in the fifth intercostal space, and the pericardial sac was cut open. The left auricle was lifted, and the mitral valve was punctured with a 20-G needle through the left ventricular free wall just below the valve location. MR was confirmed by a transthoracic echocardiography machine equipped with a 4–10-MHz phased array probe (M9, Mindray, Shenzhen, China). The MR jet area of 45–60% of the total left atrial area was considered successful and was included in the study (Fig. 1). After that, the thoracic cavity, muscular layer, and skin were closed with an absorbable suture. A similar surgical procedure was performed without puncturing the left ventricle (LV) and mitral leaflet for the sham operation. Enrofloxacin and tramadol were given daily for 7 days. All rats were allowed to develop LA and LV anatomic and molecular remodeling and altered systolic function for 8 weeks before initiating the treatment intervention.

### Experimental procedure

**Fig. 9:**
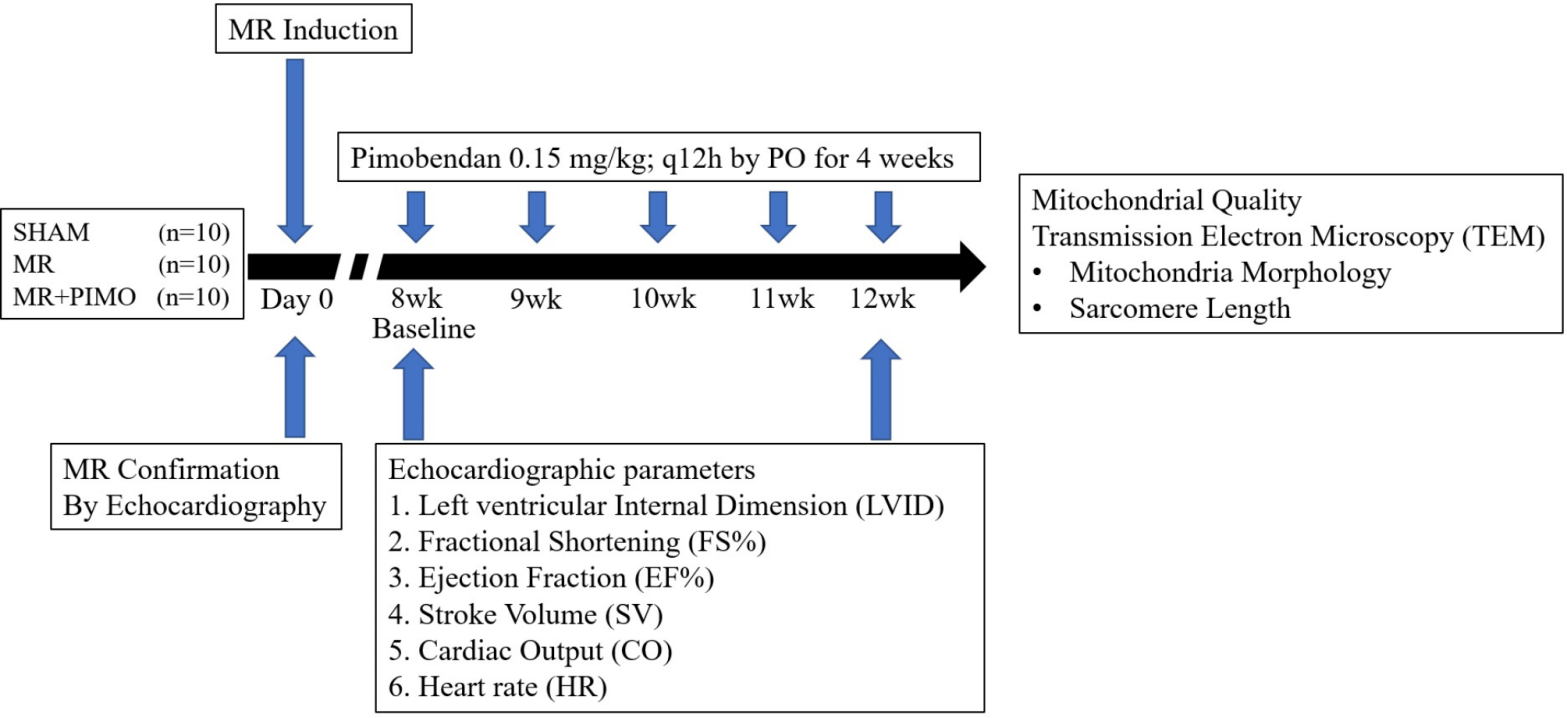
Study design to assess the effects of pimobendan (PIMO) on cardiac function, mitochondrial quality, and cardiac ultrastructure and mitochondrial morphology in rats with mitral regurgitation (MR). Eight weeks after surgical induction of MR, pimobendan (0.15 mg/kg) was given orally to MR + PIMO rats twice daily for 4 weeks, whereas MR rats received drinking water orally (5 mL/kg). Echocardiography images were obtained at baseline (8 weeks after the operation) and at the end of the study (12 weeks after treatment). At the end of the experiments, all hearts were harvested to evaluate their weight per bodyweight ratio. In addition, a heart section was processed for transmission electron microscopy to quantify mitochondrial morphology and sarcomere length. Mitochondrial quality was inferred from three mitochondrial parameters: swelling, membrane potential changes, and reactive oxygen production

Eight weeks after surgery, echocardiography was performed to confirm and document the presence of MR in the MR group, whereas no MR was visible in the sham group. The rats in the MR group were randomly divided into two groups: MR rats (n = 10) receiving drinking water 5 mL/kg, PO, q12h, and MR rats receiving pimobendan (MR + PIMO; n = 10) 0.3 mg/kg, PO, divided twice daily. The dose of pimobendan used in this study was selected based on a previous publication [43] in which chronic administration of pimobendan resulted in an increased EF in catecholamine-induced myocardial injury. Echocardiography was performed to ensure that there were no differences in echocardiographic parameters observed between the two groups at the baseline. The sham group was given drinking water (5 mL/kg, PO, q12h) as a placebo. Echocardiography was conducted again 4 weeks after therapy was initiated (Fig. 9). Four weeks after the initiation of treatment, the rats were sacrificed with an overdose of isoflurane in an anesthetic chamber to harvest their heart. Thoracotomy and vital organ removal (i.e., heart) were used as a physical confirmation of death in the current study. The heart was perfused with ice-cold phosphate-buffered saline (PBS) solution and weighed. A small portion of the LV apex (approximately 300 mg) was collected for mitochondrial isolation, whereas the trabeculae muscle of the left ventricle was obtained for TEM. The remaining part of the heart was placed in 10% formalin for 24 h, and histopathological processes were performed for hematoxylin and eosin staining.

### Echocardiography

Echocardiography was performed in all rats between 1.5 and 3.0 h post-pimobendan or placebo dosing. Rats were lightly sedated with isoflurane 2–3% mixed with oxygen through the nose cone. Images were acquired with a 4–10-MHz ultrasonic phased array probe connected to a Mindray M9 echocardiography machine, and electrocardiographic (ECG) electrodes were attached to both forelimbs and the left hindlimb and connected to the machine. Color Doppler mapping of MR jets was used to semiquantitatively assess the severity of MR. MR jet areas were assessed in the right parasternal long-axis view. LV septal and posterior wall thickness (i.e., interventricular septal at end-diastole, interventricular septal at end-systole, LV posterior wall at end-diastole, and left ventricular posterior wall at end-systole) were measured by M-mode echocardiography at the level of the head of the papillary muscle following previously published guidelines [44]. From this view, fractional shortening, EF, EDV, and ESV were calculated using the Teichholz method [45, 46]. At the base of the heart, a right parasternal short-axis view was obtained to evaluate the left atrium-to-aortic root ratio (LA/Ao). The right parasternal long-axis view with color mapping was also obtained to measure the MR jet area (%). The MR jet area (%) was quantified by the area of mosaic color observed during systole inside the left atrium divided by the total area of the left atrium and multiply by 100 which was described previously [47]. All parameters were evaluated on an average of six consecutive cardiac cycles.

### Determination of mitochondrial quality: mitochondrial ROS production, membrane potential changes, and swelling

The isolation of cardiac mitochondria was described previously [48]. Briefly, a piece of the LV apex was homogenized with an isolation buffer (300 mM sucrose, 0.2 mM EGTA, 5 mM TES; pH 7.2) in cold conditions using a Dounce homogenizer for the evaluation of mitochondrial function. A differential centrifuge was used to isolate the mitochondria. The mitochondrial pellet was re-suspended with 500 µL of ice-cold respiration buffer (100 Mm, KCl; 50 mM, sucrose; 10 mM, HEPES; 5mM, K_2_HPO_4_; pH, 7.2), and the mitochondrial protein concentration was immediately measured using the Bradford protein assay. Bovine serum albumin was used for creating a standard curve and quantifying the concentration equation.

The ROS production by the mitochondria was determined using dichloro-dihydro-fluorescein diacetate (DCFDA) in a fluorescent microplate reader [49]. The mixture of isolated cardiac mitochondria and DCFDA was prepared and added into a black microplate to obtain a final concentration of mitochondria protein of 0.4 mg/mL in a total volume of 200 µL and incubated at 37 °C for 30 min. DCFDA passed into the mitochondria and oxidized in the presence of ROS to form DCF, a fluorescent form. Fluorescence intensity was measured at excitation and emission wavelengths of λ 485 nm and λ 530 nm, respectively.

Cardiac mitochondrial membrane potential changes were determined using 5,5′,6,6′-tetra-chloro-1,1′,3,3′-tetraethylbenzimidazolcarbocyanine iodide (JC-1) [49]. The diluted JC-1 solution (5 µM) was added into a black microplate, followed by a mitochondrial solution, to obtain 0.4 mg/mL in a total volume of 200 µL and incubated at 37 °C for 30 min in the dark. Fluorescence intensities of green (JC-1 monomer) and red (JC-1 aggregate) were measured at excitation and emission wavelengths of λ 485 and λ 530 nm, and λ 485 and 590 nm, respectively. The red/green intensity ratio was calculated and represented to indicate the quality of mitochondria [50]. A reduction in the red/green fluorescence intensity indicates mitochondrial depolarization.

Mitochondrial swelling was determined by measuring the change in the optical density value at λ540 nm in the respiration buffer (mitochondrial protein concentration of 0.4 mg/mL in a total volume of 200 µL) using a microplate reader [49]. Absorbance was measured immediately and every 5 min after mitochondria were added to the buffer. A rapid loss of the absorbance measured at 30 min indicates mitochondrial swelling.

### Visualization of the myocyte ultrastructure by TEM

An LV trabeculae muscle (n = 2/group) was cut and fixed with 3% glutaraldehyde in 0.1 M phosphate buffer and stored at 4 °C for the TEM study. Then, the tissues were postfixed with 2% osmium tetroxide in 0.1 M phosphate buffer, dehydrated with an alcohol series, and embedded in epon (Epon 812; Electron Microscopy Sciences, Fort Washington, PA, USA) and polymerized at 70 °C overnight. Lead citrate and uranyl acetate were used to stain the ultrathin Sects. (65–70 nm). Mitochondrial morphology, sarcomere length, and vacuole size were visualized under a transmission electron microscope (JEM-1400 Plus; JEOL, Tokyo, Japan). Images were captured at low magnification, i.e., 2,500× for sarcomere length assessment and 10,000× for mitochondrial morphology assessment. Sarcomere lengths were measured using NIS-Elements Advance Research (Nikon, USA).

*In vitro study of the effect of pimobendan on H*_*2*_ *O*_*2*_ *-induced intracellular ROS production in H9c2 cells*.

### Cell culture and treatment

The rat ventricular myoblast cell line H9c2 (ATCC number CRL-1446) was purchased from the American Type Culture Collection (ATCC®, Manassas, USA). Cells were cultured in Dulbecco’s modified Eagle’s medium (D5030, Sigma-Aldrich) supplemented with 10% (v/v) fetal bovine serum (FBS-G1-12 A, Biocom Biotech) and 100 U/mL penicillin and 100 µg/mL streptomycin. The cells were incubated in a humidified incubator at 37 °C, under 95% air and 5% CO_2_. The medium was refreshed every 2 days. Subculture was performed when the cell density reached 80–90% confluence.

The first protocol was designed to obtain the optimum dosage of pimobendan for a subsequent study. The H9c2 cells were seeded at a density of 3 × 10^3^ cells/well in a 96-well plate until reaching 80–90% confluence. Pimobendan (Vetmedin injection, 0.75 mg/mL, MW 334.37) was freshly diluted with fresh media to several concentrations at 0.1, 1, 10, and 100 µM. These ranges of concentration were chosen as they effectively cause augmented glucose-induced insulin release in a dose-dependent manner by sensitizing the intracellular calcium in rats [51]. The cell viability assay was measured by the 3-(4,5-dimethylthiazol-2-yl)-2,5-diphenyltetrazolium (MTT) cell viability assay, which was evaluated under a microscope after incubating pimobendan for 1, 2, 6, 12, and 24 h. The dosage that did not cause 10% of the individuals to die, or lethal dose 10% (LD10), was chosen for the next protocol. In the second protocol, the effects of pimobendan on the alteration of the mitochondrial membrane potential and intracellular ROS production were determined.

### Mitochondrial membrane potential

H9c2 cells were seeded at a density of 3 × 10^3^ cells in 200 µL to a black 96-well plate. Then, 5 µM of JC-1 dye was added and incubated at 37 °C for 30 min in the dark. Fluorescence intensity was determined using a microplate reader. The fluorescence intensity for monomeric green fluorescein was determined by a fluorescence microplate reader with the excitation and emission wavelengths at 485 and 530 nm, respectively, while the aggregate red fluorescein was determined by a fluorescence microplate reader with excitation and emission wavelengths at 485 and 590 nm, respectively.

### Intracellular ROS production

H9c2 cells were seeded at a concentration of 5 × 10^3^ cells/mL in 200 µL. Cells were allowed to attach for at least 24 h. After pimobendan treatment, the media was discarded and incubated with 100 µL of complete media containing 5 µM DCFDA. The plate was incubated at 37 °C for 60 min in the dark. To determine ROS production, the fluorescence intensity was determined by a fluorescence microplate ready with excitation and emission wavelengths at 485 and 530 nm, respectively.

### Cell viability

H9c2 cells were seeded at a density of 3 × 10^3^ cells in 200 µL until reaching 80% confluence. Then, cells were incubated with a complete medium in the presence and absence of pimobendan and incubated at 37 °C. Cell viability was assessed by the MTT cell viability assay by incubation with 0.5 mg/mL of MTT dye at 37 °C for 2 h. After incubation, the MTT reagent was discarded and dimethylsulfoxide (DMSO) was added for solubilizing the formazan dye. The optical density was determined by a spectrophotometer at λ 490 nm using DMSO as a blank. The relative percentage of cell viability was compared with the control group [52].

### Determination of intracellular ROS production by H_2_O_2_ challenging

H9c2 cells were seeded at a density of 105 cells in 200 µL until reaching 80% confluence. Pimobendan was freshly prepared by diluting with the fresh media to obtain a concentration of 0, 0.1, 1, 10, and 100 µM. Two protocols were established as an ROS formation preventer and an ROS scavenger.

In the first protocol, to determine the pretreatment effect of pimobendan on intracellular ROS generation, cells were incubated with 25 µM DCFDA in a medium at 37 °C 30 min before incubation with several concentrations of pimobendan. Then, the culture medium was discarded, and cells were treated with PBS or 250 µM H_2_O_2_ in PBS for 30 min. The fluorescence signal of DCF was measured at excitation and emission wavelengths of 485 and 530 nm, respectively.

In the second protocol, for determining the ROS-scavenging properties of pimobendan, different concentrations of pimobendan were incubated with PBS (control) or H_2_O_2_ to obtain 250 µM H_2_O_2_ at 37 °C for 30 min. Before treatment, the cells were incubated with 25 µM DCFDA in a medium for 30 min. Then, the culture medium was replaced by pimobendan–PBS or pimobendan–H_2_O_2_ mixtures and incubated at 37 °C for 30 min. The fluorescence signal of DCF was measured at excitation and emission wavelengths of 485 and 530 nm, respectively. The mean fluorescence intensities are expressed as percentages of the untreated control.

### Statistical analysis

Data are presented as mean ± standard error of the mean (SEM). Heart-to-bodyweight ratios were calculated for each rat using the data at the end of the study. Statistical analysis was performed using IBM® SPSS® Statistics software (IBM Corp., Armonk, NY, USA). The normal distribution of variables was assessed using the Shapiro–Wilk test. A one-way analysis of variance (ANOVA) test with Tukey’s correction for multiple comparisons was used to evaluate the differences among groups. If the values failed to display normality, ANOVA on ranks was used to evaluate the differences among groups. *P-value* < 0.05 was considered statistically significant.

### Electronic supplementary material

Below is the link to the electronic supplementary material.

Supplementary Material 1

## Data Availability

The data underlying this article will be shared on reasonable request to the corresponding author.

## Abbreviations

ANOVA: analysis of variance
CO: cardiac output
cAMP: cyclic adenosine monophosphate
CHF: congestive heart failure
DMSO: dimethylsulfoxide
DMVD: degenerative mitral valve disease
ECG: electrocardiography
EDV: end-diastolic volume
ESV: end-systolic volume
EF: ejection fraction
H9c2: cardiac myoblasts
JC-1: 5,5′,6,6′-tetra-chloro-1,1′,3,3′-tetraethylbenzimidazolcarbocyanine iodide
LA: left atrium
LV: left ventricle
LVIDd: left ventricular internal diameter at end-diastole
LVIDs: left ventricular internal diameter at end-systole
MR: mitral regurgitation
MTT: 3-(4,5-dimethylthiazol-2-yl)-2,5-diphenyltetrazolium
PBS: phosphate buffered saline
PDE-III: phosphodiesterase-III
PIMO: pimobendan
ROS: reactive oxygen species
SEM: standard error of the mean
TEM: transmission electron microscope
