# Supplementary material for: Pimobendan prevents cardiac dysfunction, mitigates cardiac mitochondrial dysfunction, and preserves myocyte ultrastructure in a rat model of mitral regurgitation
Source: BMC Vet Res. 2023 Aug 23;19:130. doi: 10.1186/s12917-023-03693-2 (PMC10463781; doi:10.1186/s12917-023-03693-2)
Supplement: Supplementary file 1 — Supplementary Material 1 [file 12917_2023_3693_MOESM1_ESM.docx]

Supplement Table 1 List of echocardiographic parameters and statistic report obtained from anesthetized rats at baseline.


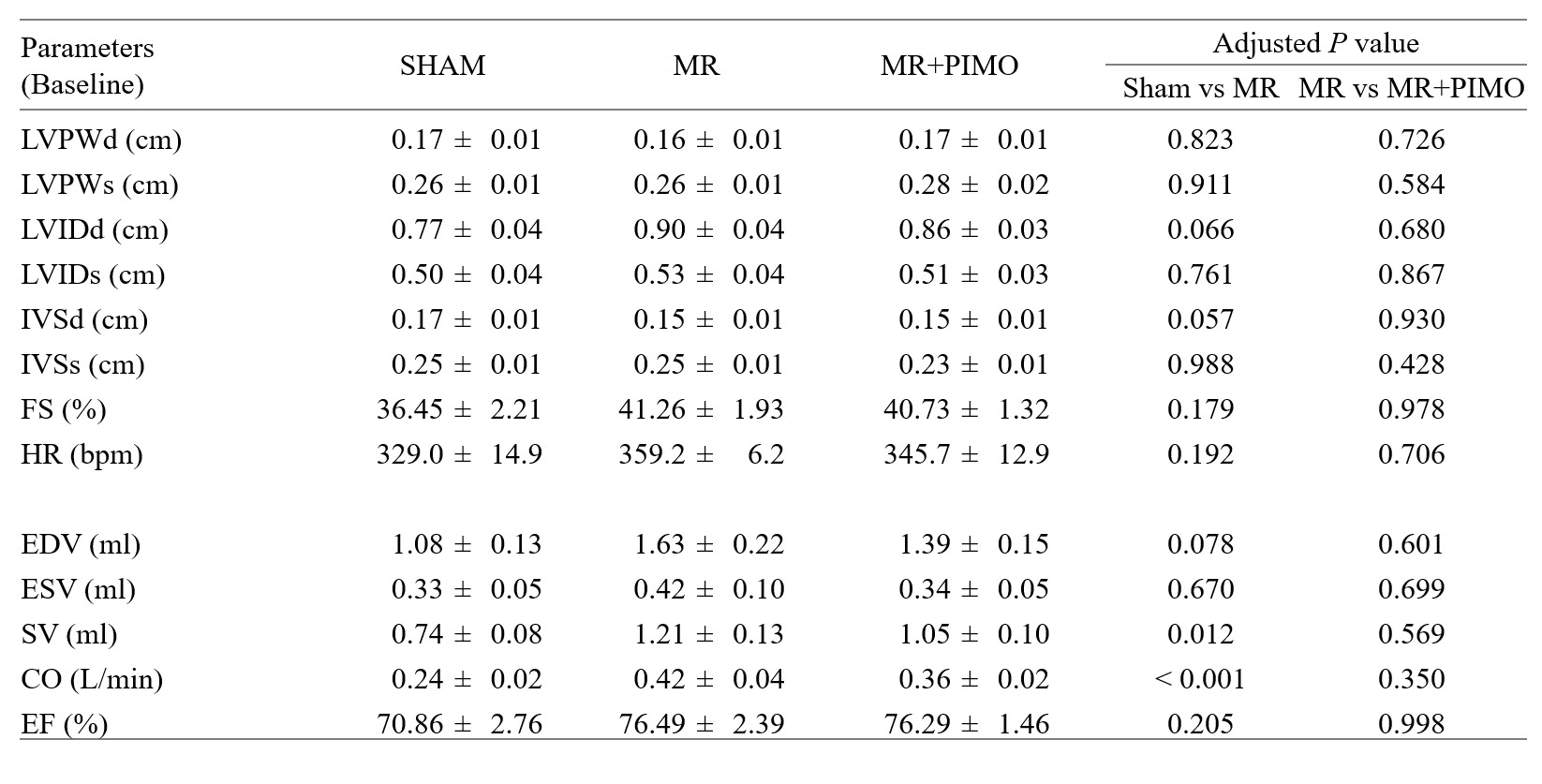


Data are presented as mean ± standard error of the mean (SEM). Statistical analysis was performed using IBM^®^ SPSS^®^ Statistics software (IBM Corp., Armonk, NY, USA). A one-way analysis of variance (ANOVA) test with Tukey’s correction for multiple comparisons was used to evaluate the differences among groups. CO: cardiac output; EDV: end-diastolic volume; ESV: end-systolic volume; EF: ejection fraction; FS: fractional shortening; HR: heart rate; IVSd: interventricular septal at end-diastole; IVSs: interventricular septal at end-systole; LVIDd: left ventricular internal diameter at end-diastole; LVIDs: left ventricular internal diameter at end-systole; LVPWd: left ventricular posterior wall at end-diastole; LVPWs: left ventricular posterior wall at end-systole; MR: mitral regurgitation; PIMO: pimobendan; SV: stroke volume

Supplement Table 2 List of echocardiographic parameters and statistic report obtained from anesthetized rats at the end of the study.


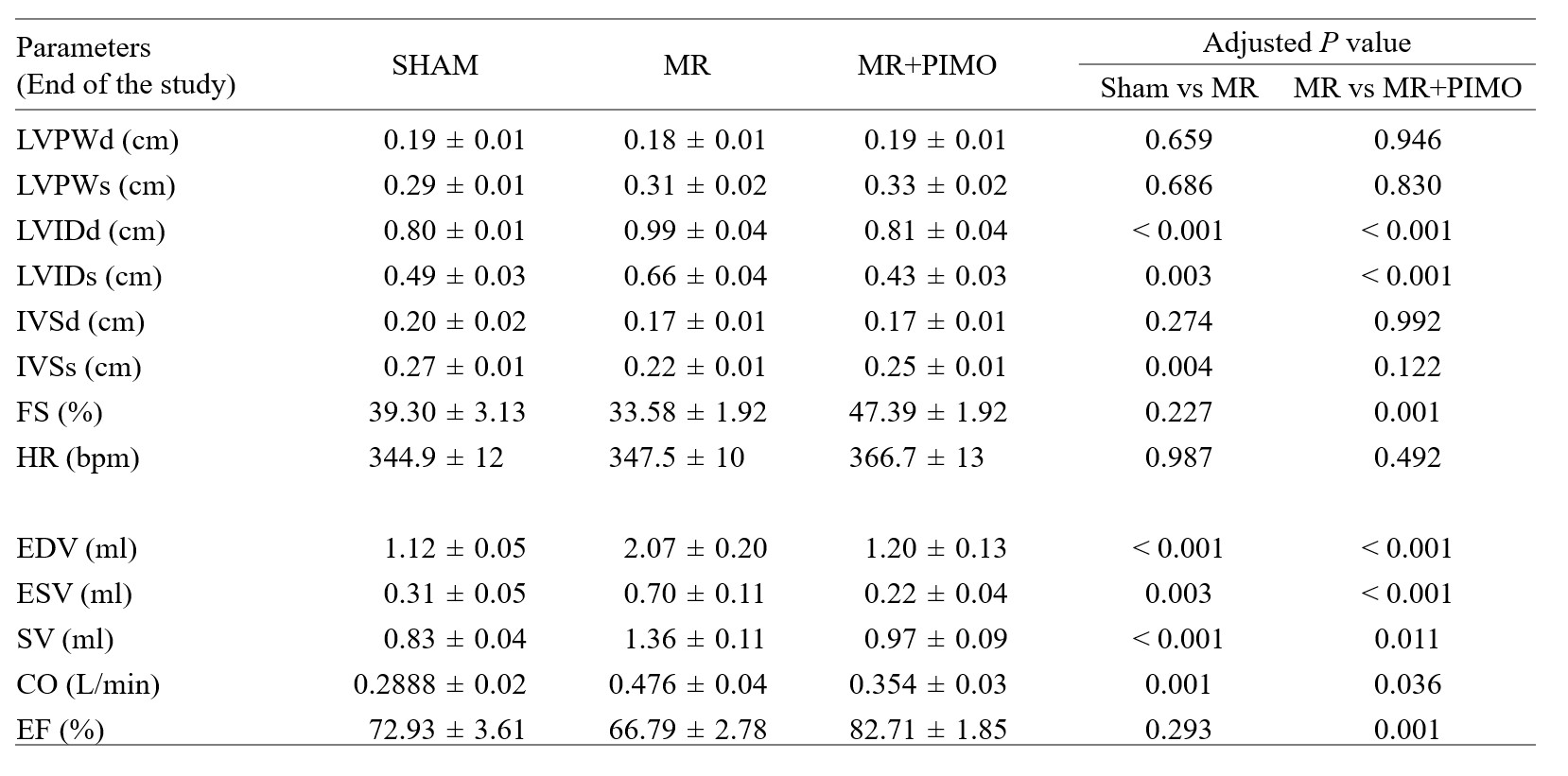


Data are presented as mean ± standard error of the mean (SEM). Statistical analysis was performed using IBM^®^ SPSS^®^ Statistics software (IBM Corp., Armonk, NY, USA). A one-way analysis of variance (ANOVA) test with Tukey’s correction for multiple comparisons was used to evaluate the differences among groups. CO: cardiac output; EDV: end-diastolic volume; ESV: end-systolic volume; EF: ejection fraction; FS: fractional shortening; HR: heart rate; IVSd: interventricular septal at end-diastole; IVSs: interventricular septal at end-systole; LVIDd: left ventricular internal diameter at end-diastole; LVIDs: left ventricular internal diameter at end-systole; LVPWd: left ventricular posterior wall at end-diastole; LVPWs: left ventricular posterior wall at end-systole; MR: mitral regurgitation; PIMO: pimobendan; SEM: standard error of the mean; SV: stroke volume

Supplement Table 3 List of statistic report of percentage of cellular viability obtained from H9c2 cells treated with several concentrations of pimobendan **(**0, 0.1, 1, and 10 µM**)** at any of the incubation times **(**1, 2, 6, 12, and 24 h**)**. The upper table demonstrates percentage of cellular viability while the lower table shows p-value between groups.


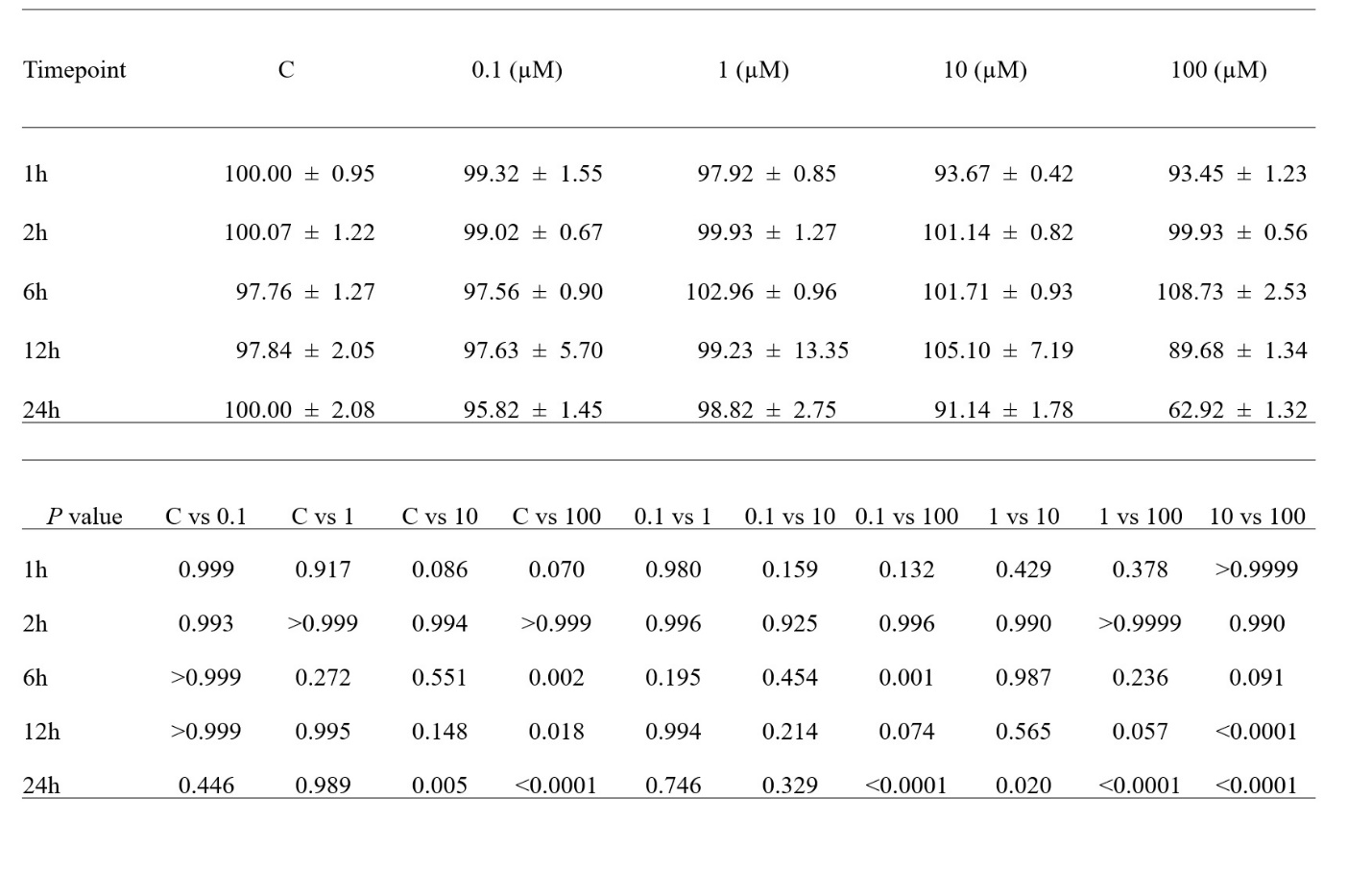


Data are presented as mean ± standard error of the mean (SEM). Statistical analysis was performed using IBM^®^ SPSS^®^ Statistics software (IBM Corp., Armonk, NY, USA). A two-way analysis of variance (ANOVA) test with Tukey’s correction for multiple comparisons was used to evaluate the differences among groups.
